# Managers' perceptions of the manager role in relation to physicians: a qualitative interview study of the top managers in Swedish healthcare

**DOI:** 10.1186/1472-6963-10-271

**Published:** 2010-09-17

**Authors:** Mia von Knorring, Angelique de Rijk, Kristina Alexanderson

**Affiliations:** 1 Division of Insurance Medicine, Department of Clinical Neuroscience, Karolinska Institutet, Stockholm, Sweden; 2 Department of Social Medicine, Faculty of Health, Medicine and Life Sciences, Maastricht University, Maastricht, The Netherlands

## Abstract

**Background:**

This study focused on the manager role in the manager-physician relationship, considered from the manager perspective. The aim was to understand how top executives in Swedish healthcare regard management of physicians in their organisations, and what this implies for the manager role in relation to the medical profession. Abbott's theory of professional jurisdiction was used to inform thinking about managerial control and legitimacy in relation to physicians.

**Methods:**

Data from semi-structured individual interviews with 18 of the 20 county council chief executive officers (CEOs) in Sweden were subjected to qualitative analysis.

**Results:**

The results show that, when asked about their views on *management *of physicians, the CEOs talked about "how physicians are" rather than describing their own or their subordinate managers' managerial behaviour or strategies. Three types of descriptions of physicians were identified: 1) they have high status and expertise; 2) they lack knowledge of the system; 3) they do what they want in the organisation. The CEOs seldom reported that general management strategies were used to manage physicians. Instead, they described four types of physician-specific management strategies that were used in their organisations: organisational separation of physicians; "nagging and arguing"; compensations; relying on the physician role. These strategies seemed to reflect pragmatic behaviour on behalf of the managers that helped them to maintain control over physicians in daily work. However, in a longer perspective, they seemed to decrease the legitimacy of the manager role and also contribute to weakening of that role in the organisation.

**Conclusions:**

Many CEOs seemed to regard the manager role in their organisations as weak and described difficulties in both taking and defining that role (for themselves or others) in relation to the physician role. Further research is needed to elucidate how managers in healthcare organisations assume the manager role in relation to the medical profession. Studies indicate that lack of clarity concerning manager role authority and responsibility may have negative consequences not only for the working conditions of managers, physicians, and other healthcare professionals, but also for the quality of care.

## Background

"Oh [management of physicians]—I could talk about that forever..."

This is a quotation from an interview with a manager holding one of the highest executive positions in the Swedish healthcare system. Why is it that this manager, who is probably very busy and has all the formal power that can be obtained within the organisation, feels that he or she could talk forever about managing physicians?

Difficulties in managing healthcare organisations have long been documented [1-3], which is hardly surprising, considering the complexity of such systems. It has been suggested that managerial problems in healthcare should instead be viewed as a basic condition [4] and that research should not concentrate on identifying problems, but should be aimed at explaining the how's and why's of the difficulties in order to offer a basis for improvement. In this context, it has been proposed that leading and managing physicians, unlike managing other healthcare professionals, may put specific demands on managers in healthcare organisations [5]. The manager-physician relationship has been pointed out as a critical determinant of the success of healthcare organisations [6]. However, problems in this relationship have been reported by both managers and physicians in many Western countries, and the need to improve the situation has been addressed in a large number of studies and editorials over the last decades [3,7-11].

The present study focused on the role of the manager in the manager-physician relationship. In this article, the term "manager" designates a person employed in a managerial position, regardless of the level. However, it is important to begin by making a clear distinction: our objective was not to describe the behaviour of individual managers nor to illustrate physicians or physician's behaviour, but rather to study the *manager role *that is defined as a social role accompanied by specific responsibilities, disregarding the individuals who during a certain period hold that role [12].

Abbot's sociological theory of professional jurisdiction [13] informed our study. According to Abbott, jurisdiction is the link between a profession and the content of its work tasks, and in this respect it is not an issue of legal formulations or specific work descriptions, but a process that is created and experienced in work, anchored by formal and informal social structures. Jurisdiction is continuously claimed and negotiated in daily work between different professions or occupations, and strong (i.e., established) professions assume more power than weaker professions or occupations [13].

The medical profession is well established and represents one of the oldest professions in society. Abbott has analysed why this profession is strong, not only from the perspective of how physicians are trained and organised, but also in terms of jurisdiction. Physicians are generally responsible for an obvious, legitimate area of medical tasks that is rarely disputed by other professionals [13]. By comparison, managers' jurisdiction over their tasks is not that self-evident. The role of manager in healthcare organisations is a relatively new phenomenon and cannot be regarded as a profession in a classical sense [13,14]. Historically, this role has also been closely connected to the medical profession; that is, most managers have been physicians by training. However, in recent decades, separation of the manager and physician roles has begun, and in many countries this process has been strongly reinforced by government policy [6]. In 1983, Sweden introduced the first law that allowed professionals other than physicians to take administrative responsibility for hospitals and clinics. In 1997, the formal separation of the two roles was completed, when the role of clinical manager (involving direct administrative management of practicing physicians and other care professionals) also became open to professionals other than physicians [15]. These laws have paved the way for a potential manager professionalisation process in healthcare organisations and for a strengthening of the manager role in relation to the medical profession. However, several studies have shown that this process easily fails and have addressed the difficulties that managers encounter in relation to sustaining a process of professionalisation [13,16,17]. For example, Salter [18] observed that increased managerialism did not increase the control over physicians; this investigator analysed the situation in the United Kingdom (where there is extensive government power over healthcare) and found that the strong medical profession tends to criticise increased management and use effective tactics to remain independent. Similarly, a study conducted in the Netherlands [19] demonstrated how legislation regarding disability pension was not put into practice, because the physicians opposed it, which shows that those professionals were such influential advocates that they had sufficient autonomy to decide how new rules should actually be applied.

According to Abbot [13], jurisdiction can be claimed in three different arenas: the legal, the public, and the workplace arena. This paper focuses on the workplace arena, in which Swedish healthcare organisations can be described as professional bureaucracies with varying degrees of divisionalisation [20]. In this type of organisation, managers have instrumental power and authority that are based on their managerial position [21], but, simultaneously, the professionals at operative levels possess a large amount of autonomy and independence.

*The aim *of this study was to understand how the top managers in Swedish healthcare regard management of physicians in their organisations and what this implies for the managerial role in relation to the medical profession.

## Methods

Data collected in semi-structured individual interviews with county council chief executive officers (CEOs) were subjected to qualitative analysis [22].

### Participants and setting

Sweden has a population of nine million, and the responsibility for delivery of healthcare to the population is organised through 20 county councils. The county councils have populations ranging from 100 000 to almost two million. At the time of the study, the number of employees in the county councils ranged from approximately 4000 to 46 000, of which 400 to 4000 were physicians [23].

Each of these county councils is supervised and coordinated by a CEO. The CEOs are appointed by the government of their county councils and are the highest executives in the Swedish healthcare system, with an overarching responsibility for hospitals as well as other healthcare services. These leaders are responsible mainly for economic and strategic issues, including the tasks of formulating visions, policymaking, goal setting, and communicating these strategies to all parts of the organisation. The CEOs do not manage physicians directly; their role is to manage managers, and they run their organisations through subordinate managers in a line management system. Depending on the size of the county council, there can be from one to several managerial levels (usually several) between the CEOs and the practicing physicians in the organisation.

All 20 of the county council CEOs in Sweden were invited to take part in this project. Two chose not to participate and referred us to subordinate managers, but the interviews with those lower-level managers were not included in this study. In all, data from individual interviews with 18 (90%) of the CEOs were included. The participants represented both large and small county councils in rural as well as urban areas. Seven of them were trained as physicians, whereas the remaining 11 had other professional backgrounds. Five were women.

The study was part of a larger research project on how managers in healthcare organisations manage the process of sickness certification of patients who are unable to work due to illness or injury. This e.g. includes management strategies for competence development regarding sickness certification processes, strategies for cooperation within healthcare and with other stakeholders regarding these issues, and for quality assurance of related processes [24].

### Data collection

In 2006, a letter was sent to the CEOs inviting them to participate in individual interviews concerning leadership and management of the process of sickness certification of patients in their organisations. Most of the interviews were conducted by phone, although personal meetings were held when possible. Two experienced interviewers who had also worked with management issues in healthcare carried out the interviews. All participants were informed that they had the right to withdraw from the study at any time. Each interview lasted approximately 45 minutes and was recorded in MP3 format and transcribed verbatim. Each interview transcript consisted of 15-30 typed pages. The validity of the transcripts was checked by parallel listening and reading through one third of the 18 interviews.

Initially, an interview guide that included open-ended questions concerning CEOs' views on management of physicians in relation to the task of sickness certification was tested by conducting two interviews with managers who had previously had positions as CEOs. This pilot study showed that the interviews provided extensive information about the managers' experiences in managing physicians and that the respondents to a large extent seemed to describe general aspects regarding management of physicians. Therefore, a question that focused specifically on the CEO's views on management of physicians in general was added to the guide. This query was phrased as follows: "Could you please tell me about your views on management of physicians?" The broad concept "management" was not defined in the interviews in order to induce the CEOs to explore as many aspects of this issue as possible and to do so from the perspective of their own pre-understanding of management.

### Data analysis

The data analysis was loosely based on a grounded theory approach as described by Corbin and Strauss [25], and it was performed in three steps: initial open data exploration, followed by identification of the concepts and their relationships, and finally development of a story line. In the first step, the interview transcripts were scrutinised by the first author, and all statements mentioning physicians were extracted. Those assertions were then gathered in one document, which was read several times to get a sense of the whole. Next, to form the unit of analysis for the study, the first two authors identified all statements that expressed views concerning management of physicians. The texts on management of physicians that were compiled from each interview varied in length from a few sentences to more extensive formulations of several pages. Thereafter, the content of each statement (meaning unit) was condensed, and each condensed meaning unit was given a code following the procedure reported by Graneheim and Lundman [26] (see Table 1). During the next stages of analysis, the empirically grounded findings were related to, and integrated with, prior theory and the authors' own pre-understanding [27,28]. All types of associations with both prior and emerging theories were continuously written down in memos by the first author [25], who then discussed the identified memos, codes, categories, and themes with the other authors, who searched the text for second opinions. Concepts and their interrelationships were subsequently developed in discussion with all authors. In parallel to this step of the analysis, a story line was developed in which the concepts found in the analysis were represented in relation to each other [25].

**Table 1:** An example of the analytical procedure: from meaning unit to code

Meaning unit	Condensed meaning unit	Code
"In the first place, there's a culture /.../that allows you to do what you want. It's an accepting culture, a permissive culture, there's very large variation. And it's probably a, a matter of course to some extent, of loyalty to colleagues and solidarity, to..., yes, you allow such extremely extensive variation."	A culture that is accepting, permissive, and loyal, and allows physicians to do what they want.	"Physicians can do what they want"

In the results section, identified codes and themes in the statements are illustrated by direct quotations from the interviews, in which/.../indicates that text has been omitted, and [] shows that text has been added. All additions and omissions of text were done for practical reasons and did not change the connotation of the quotations. All quotes presented here were translated from Swedish. Also, they can be related to a specific interviewee by identification letters given within parentheses; for example, (A.P) if the interviewee is trained as a physician and (A.nP) if the interviewee is not trained as a physician.

The study was approved by the Regional Ethical Review Board of Stockholm.

## Results

We identified two categories of statements about how the CEOs regarded management of physicians in their organisations (Table 2). The first category concerns descriptions of physicians' behaviour (I), and the second comprises descriptions of strategies to manage physicians (II). Below, we first describe those categories and then analyse their implications for the manager role in relation to the medical profession (III).

**Table 2:** Identified categories and subcategories of top managers' descriptions regarding physicians and strategies for managing physicians.

I. Descriptions of physicians	II. Strategies to manage physicians	
1. "Physicians have high status and expertise"	A. General management strategies	B. Physician-specific management strategies
	
2. "Physicians lack knowledge of the system"	1) Management control 2) Motivational strategies 3) Line management	1) Organisational separation 2) Nagging and arguing 3) Compensations 4) Relying on the physician role
3. "Physicians can do what they want"		

### I. Descriptions of physicians

Even though the interviews clearly focused on *management *of physicians, nearly half of the statements did not deal with the managers' role in the manager-physician relationship. In short, rather than addressing their own or their subordinate managers' managerial behaviour in relation to physicians, these statements merely contained descriptions of physicians' behaviour, as perceived by the CEOs. The views on the behaviour or characteristics of the physicians were formulated either in relation to them as individual professionals, as a professional group, or in terms of their professional culture. Three types of statements were identified.

#### 1. "Physicians have high status and expertise"

One type of statement concerned physicians' status in the organisation. The CEOs clearly acknowledged physicians' medical expertise and academic competence, and described them as a professional group of high standing in the organisation (i.e., with high social status among healthcare professionals). However, this status was also criticised, and some felt that physicians almost expected admiration from others in the organisation.

[Management of physicians] is a stimulating job, of course, you can't say otherwise. But it's demanding, it's about talented, highly educated people who have a university education, they have a high status and are used to being admired. (G.nP)

In such statements, the demands of managing physicians were not associated with the managers' strategies or behaviour, but rather with the physicians' high standing in the organisation.

#### 2. "Physicians lack knowledge of the system"

A second type of statement concerned physicians' organisational knowledge and competence. CEOs described physicians as lacking knowledge of the system in which they work, not only with respect to the healthcare organisation per se, but also regarding the role of healthcare in society.

What really surprises me sometimes is physicians' very inadequate understanding of the system, how limited they are in their world. I mean, they're extremely knowledgeable in their field, but they don't—maybe because they haven't cared or they haven't regarded it as necessary or they haven't had the time, I haven't got the slightest idea—but they really don't know much about the system they're part of. And under what conditions I [as CEO] function, why we have politicians, that there are other sectors that it's important for me to influence in my work. That has really surprised me. (D.nP)

Another CEO formulated it like this:

Physicians are trained to consider the needs of the individual patient, they are trained to solve issues on their own, but they are less trained in teamwork, to belong to a team, and take a societal perspective. And that's difficult, both for the physicians, but it's hard for us as management, and it's often that, I suppose, which leads to frustration for both parts (C, nP)

According to this CEO, the lack of system knowledge affects the ability of physicians to cooperate and therefore makes them a difficult group to manage.

#### 3. "Physicians can do what they want"

A third type of statement concerned physicians' autonomous behaviour in the organisation. CEOs described how physicians tended to avoid participating in meetings with other professional groups, were reluctant to abide by rules, and in different ways chose to follow their own agendas. This type of "do-what-you-want" behaviour was perceived as strong and not limited to medical issues.

They very much guard how they exercise their own professional practice. That they have the preferential right of interpretation, that it is not the deliverer of care, from some holistic picture, who has the preferential right of interpretation, but rather it is the individual physician who has that in all situations, not only in the direct consultation with the patient where you make an assessment, but in all matters (B.P).

Physicians' autonomous behaviour in the organisation was not argued as a consequence of the CEOs' or their subordinate managers' decisions or strategies. Instead, it was attributed to a strong collegial culture among physicians that was described as being "permissive" and based on loyalty and solidarity within the medical profession.

There is a culture/.../that allows you to do what you want. It is an accepting culture, a permissive culture/.../yes; you allow such extremely extensive variation. (B.P)

Some CEOs mentioned a general antipathy among physicians towards external pressure and influence. This antipathy was attributed to physicians' professional culture, which was described as being responsible for forming physicians' views on managers and management.

This long upbringing, which medical training indeed is, in handling much individual, autonomous work makes some physicians feel that we—and that means not only me as manager, but the whole surrounding world—come and mess up their lives by not letting them do their work. (C, nP)

Such statements also indicate that the perceived difficulties in managing physicians are associated with the physicians' behaviour. In short, that it is the strong professional culture that allows physicians to "do what they want", not the managers' strategies or behaviour.

### II. Strategies to manage physicians

Notably, even though the majority of the CEOs described management of physicians as a difficult task (e.g., they frequently used expressions such as "it's frustrating", "physicians are demanding", and "it's the most difficult group to manage"), only half of the statements concerned strategies specifically aimed at managing physicians. Two subcategories of such strategies were identified: general management strategies and physician-specific management strategies.

### A. General management strategies

A few CEOs described general management strategies, which were not oriented specifically towards physicians and seemed to be based on the assumption that every professional group, in healthcare or elsewhere, requires a specific approach from the manager.

Yes, [managing physicians] requires some special finesse. But I mean, I don't think that physicians are different than any other what you might call super pros./.../they're a very specialised group with extensive in-depth knowledge. But I've worked in, for example, [another area of business] before, and it's the same there. (I.nP)

Three types of general management strategies were identified.

#### 1. Management control

One type of statements concerned different management control systems (e.g., balanced score card) that were used as a management instrument in the organisation and applied to all professionals, not just physicians.

Yes, it [managing physicians] is like [managing] all professional groups. I suppose it's the same at Ericsson and everywhere./.../It's a part of management control (E.P).

#### 2. Motivational strategies

Another type of statements in this subcategory concerned different kinds of motivational strategies aimed at influencing subordinate behaviour. One example of this was trying to implement the use of evidence-based methods by making physicians participate in various types of quality registers.

We work hard to implement evidence-based care/.../[and] guidelines, but it is difficult. It takes time before anything happens/.../. I mean, if you have very creative and active co-workers, they will create both their own judgements and norms, which may not always be all that scientific/.../. Therefore, what we say now is that they should participate in the quality registers we have/.../and be able to demonstrate how they, based on knowledge acquired from the registers, use it in clinical practice (N.P).

#### 3. Line management

A few statements explicitly addressed line management and the manager role in relation to physicians. In those cases, the CEOs clearly stated that their own role was to manage managers and that it was the responsibility of their subordinate line managers to manage physicians. For example, a need to limit physicians' freedom was argued as a clear line-manager responsibility.

You have to be aware that you, as a line manager, have to standardize and limit individual freedom. And that applies to/.../all/.../questions concerning treatment of patients./.../That is probably why this issue [management of physicians sickness certification practice] gets so, well, I won't say fraught with conflict, but to some extent that is actually the case, because this restricts the physicians' degree of freedom. (M.nP)

### B. Physician-specific strategies

The absolute majority of the statements in this category, however, concerned strategies used *specifically *to manage physicians. These strategies seemed to be completely independent from those that applied to other professionals, and they were specifically adjusted to parry or handle the difficulties that the CEOs perceived concerning physician behaviour. Four types of physician-specific strategies were identified:

#### 1. Organisational separation

One type of management strategy in this subcategory was to separate physicians from other professionals in the organisation. An example of this was to have separate department meetings for physicians even when the issues to be discussed concerned the whole staff. This strategy, the CEOs argued, was necessary to make physicians attend the meetings at all.

It is generally difficult to get physicians to attend the same ward meetings as other staff members. And that is a problem/.../because it means that to be able to get physicians to take part in such gatherings you almost have to arrange special meetings, to make it seem like a meeting that you're called to and where you can conduct a reasonable discussion." (G.nP)

#### 2. "Nagging and arguing"

Another management strategy seemed to consist of a "nagging and arguing" behaviour in which the CEOs repeatedly try to tell physicians what they should do and what their responsibilities as employees are. One CEO reported:

...if not every day, so at least very often I meet physicians and try to explain to them what their full responsibilities are/.../. They have a legally stipulated responsibility in relation to their patients, yes, but they also work for us, either under a contract/.../or they are employed by us and are supposed to fulfil our requirement of working in an evidence-based manner. (K.nP)

However, some of the CEOs argued that repeated reference to rules and regulations was not an effective strategy to manage physicians.

#### 3. Compensations

A third management strategy was to compensate physicians for participating in activities or meetings that the manager regarded as important. Such compensation was not related to ordinary salary or negotiated agreements or privileges, but was instead specifically offered by management in an effort to make participation in a particular activity attractive to physicians. One CEO formulated it like this:

It gives us some degree of freedom to be able to put in some money and "sweeten" some activities that physicians traditionally do not prioritize. After all, this is very much about selling a message to the physicians/.../. There's always a discussion when physicians are supposed to participate in training activities—how much can it cost? For this [a competence development activity concerning sickness certification] we offer [each physician] 1000 Euros just like they do in county council X. We have also discussed compensation for the physicians who use their working hours to participate in collaboration meetings/.../I think you have to be a little wise and pragmatic here. This is not about bribes, but rather about reasonable incentives and stimulation (L.P)

Another CEO was more hesitant with regard to this type of compensation, but argued that some kind of extra compensation is necessary to manage physicians:

We have also discussed whether to, yeah, you could say use some kind of compensation/.../how can we compensate or motivate these physicians to participate in the meetings with the Social Insurance officers that are supposed to be held within three months of the date a patient is given a sick note. There has been some discussion about paying for it as an extra compensation to the physicians, but we were a little hesitant about that/.../We think we will [instead] put it [the money] in some type of competence, what do you call it, competence development account for the individual physician [to use]. Because the point is not that they should have it as extra salary, because then... we are a little hesitant about that/.../. but to put it [the money] in an individual competence development account, that's what we'll do (P.P)

Characteristic of the content of the various forms of compensation offered to the physicians was that it was not related to the content of the activities for which they received compensation. Moreover, compensation was given for activities that, from the managers' perspectives, were part of the physicians' ordinary work obligations and were performed during the physicians' normal working hours.

#### 4. Relying on the physician role

A fourth management strategy was to rely on the physician role instead of the manager role when it came to managing physicians. This was applied in a number of ways and was reported both by CEOs who were trained as physicians and by CEOs who had other training. An example of this strategy was to identify an influential non-manager physician in the organisation who was willing to "fight for the ideas" that the manager wanted to pursue, and then "hope" that this physician would make other physicians follow. One CEO put it like this:

This [management of physicians] is about finding out what knowledge there is and when people will be prepared to fight for these ideas and [then] use those people in the context at hand, and hopefully colleagues will follow too. (G.nP)

Another approach was to rely on *the physician in the manager role*, that is, the physician role of those managers in the organisation who were also trained as physicians. Many of the CEOs, both those who were themselves physicians and those who were not, argued that it was easier for managers who were trained as physicians to control physicians' behaviour. Managers therefore tended to rely on this physician role, their own or that of subordinate managers, in managing physicians. This strategy seemed to be based on the assumption that the manager role was not strong enough to manage physicians. It was *the physician *in the manager role, not the manager role in itself, that was perceived as affecting physician behaviour.

### III. Implications for the manager role

**Figure 1:**
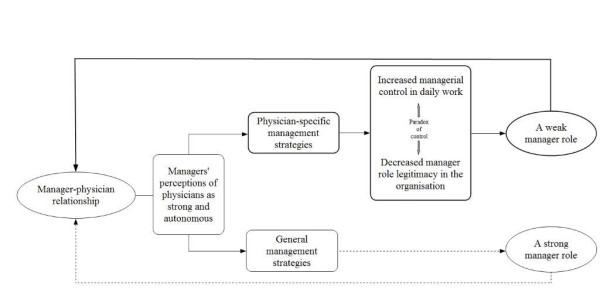
**Potential strengthening and weakening pathways for the manager role**. The diagram illustrates the relationship between healthcare managers' strategies to manage physicians and pathways that potentially strengthen and weaken the manager role in relation to the medical profession in healthcare organisations.

Our results show that the CEOs had a strong focus on the physician part of the manager-physician relationship. The medical profession was perceived as strong and autonomous by all the CEOs, and they described two different subcategories of strategies to manage them: general management strategies and physician-specific management strategies. In the following sections, we address the implications of these strategies for the manager role in relation to the medical profession. The relationships between the different strategies and the manager role are depicted in Figure 1.

#### General management strategies

The results indicate that general management strategies may strengthen the manager role in relation to the medical profession. CEOs that mentioned general strategies described confidence in the manager role, and they referred to the managerial responsibility in that role, regardless of the manager's basic profession. These were actually the only statements that included clear declaration of a strong manager role in relation to the medical profession. One CEO formulated it like this:

All those issues have to be handled in line management./.../The important thing is that you can put this on the agenda of every line manager. (K.nP)

However, few of the CEOs' statements included mention of general strategies used to manage physicians, and therefore the possible implication of such strategies is illustrated by only a dotted line in Figure 1.

#### Physician-specific management strategies

Most statements referred to management strategies that were specific for physicians. However, it seemed that those strategies did not increase the managerial jurisdiction of the managers, but rather contributed to a weakening of the manager role in the organisation. This is given further consideration in the following section and is illustrated in Figure 1.

##### Increased managerial control in daily work

The physician-specific management strategies seemed to be based on pragmatic behaviour on behalf of the managers in the organisation. The CEOs described these strategies as "necessary" to make physicians "take part in the system at all" or to prevent them from "doing what they want" in the organisation. The strategies seemed to serve the main purpose of preserving good relations with the physicians, while maintaining a certain degree of manager control, and in this respect they contributed to increased managerial control over physicians in daily work. One example of this concerns the strategy of "relying on the physician role", as illustrated by the following:

It is where we have had physicians in management, physicians who lead and manage; it is there we have been able to have good interplay. What we achieve then is to eliminate problems, and you create prerequisites to have control over daily life/.../and that also leads to a better working environment. (C.nP)

##### Decreased manager role legitimacy in the organisation

However, in a longer perspective, those physician-specific management strategies seemed to decrease manager role legitimacy in the organisation. Several CEOs addressed this dilemma. For example, it was argued that the strategy of "organisational separation" further lowers physicians' sense of belonging in the system.

I think that the decisive point in this context is that we [CEOs] have to understand that physicians are included in an organisation as a whole, and thus they do not constitute their own part of the healthcare system./.../I think/.../that applies in general, to succeed in this work, the physicians have to be involved in it, yeah, maybe even have to be forced to actually be part of a unified organisation. (M.nP)

Another example concerned the strategy of nagging and arguing that was pointed out as undermining manager legitimacy and contradicting its purpose, in a longer perspective.

It's hard to manage physicians/.../to stick to rules and strict criteria and things like that, because physicians always try to understand why, and if they're not completely convinced about the motives, they "kind of do what they want to". (F.P)

##### A weak manager role

Thus it appears that the physician-specific management strategies lead to a paradox of control in relation to the medical profession. At the same time as they increase managerial control in daily work, they seem to decrease the managers' role legitimacy and contribute to a weakening of the manager role in the organisation. The struggle for receiving legitimacy as a manager in relation to physicians was discussed specifically by several of the CEOs, both by those who were themselves physicians and by those who had other types of professional training. One CEO formulated it like this:

When it comes to management of physicians, there are strong demands on the person doing the supervising, with regard to legitimacy in that role./.../it's not all that easy/.../to match yourself—which you have to do in contexts like this—with physicians. It's hard. Actually, often physicians don't recognise anyone but a physician as a manager. (G.nP)

Another CEO stated:

I have seen a number of cases of successful and unsuccessful leadership during my years in the county council. And more recently, being in charge, unfortunately I have also seen that it is difficult for any categories other than doctors to supervise doctors. That's the way it is. We have had several nurses in high positions, also as CEOs, and it hasn't worked out very well. It's hard. (L.P)

As illustrated by these statements, the manager role is regarded as weak in relation to the medical profession. However, it seems that this weak manager role is not based solely on the relationship between managers and practicing physicians, but is also reinforced by how the CEOs perceive their own manager role, as well as that of other CEOs or subordinate managers. These top-level managers actually seem to feel that the manager role in itself does not have enough power to enable management of physicians. Therefore, the managers have to rely on the stronger physician role in the organisation, their own or that of others, when managing physicians.

## Discussion

This study was based on interviews with 18 of the 20 county council CEOs in Sweden. The aim was to understand how the top managers in the Swedish healthcare system regard management of physicians in their organisations and what this implies for the manager role in relation to the medical profession. Most of the participating CEOs said they found it difficult to manage physicians, but when asked about their views on *management *of such professionals, half of their statements merely contained descriptions of "how physicians are" rather than descriptions of their own or their subordinate managers' managerial behaviour or strategies. Three types of views concerning physicians were identified: (1) they have high status and expertise; (2) they lack knowledge of the system; (3) they do what they want in the organisation. When management was described, only a few statements concerned the use of general management strategies in relation to physicians. Instead, four types of physician-specific management strategies were described: organisational separation of physicians; nagging and arguing; compensations; relying on the physician role. The physician-specific strategies helped managers to maintain control over physicians in daily work but, in a longer perspective, seemed to decrease manager role legitimacy and contribute to a weakening of the manager role within the organisation.

Using Abbott's terms [13], the CEOs' views and strategies identified in this study are part of how managerial jurisdiction is negotiated in the workplace arena between the medical profession and the manager role. However, our results show that this negotiation takes place not only between formal managers on one side and practicing physicians with no formal manager responsibilities on the other, but also *between managers*, both laterally (between managers on the same level) and vertically (in relation to subordinate managers). Even though several studies have addressed problems in the relationship between managers and practicing physicians [3,7-11], the research in this area is limited from the perspective of managers' own role taking. CEOs in Swedish healthcare run their organisations through subordinate managers in a line management structure, and they do not personally manage physicians. Accordingly, the CEOs in this study were asked about their views on *management *of physicians in their organisations. However, even though the CEOs had the highest possible managing position in their organisations, many of them argued as if they were actually the immediate managers of the physicians. They gave detailed and sometimes emotional descriptions of physicians' behaviour and the strategies used to manage them. Of course this might indicate that managers at subordinate levels in the organisation experience the task of managing physicians as very troublesome, and that this perception is also conveyed to the highest levels in the organisation. However, in this study we found that the CEOs, when they talked about management of physicians in their organisations, often tended to focus and rely on the physician role, rather than the manager role. We found that this was done in a number of ways, in relation to not only practicing physicians, but also colleague CEOs and subordinate managers. Many of the CEOs actually seemed to regard the manager role (their own and others') as weak in relation to the medical profession, and described management strategies that contributed to further weakening of that role.

Our findings rather indicate a lack of clarity concerning how to take the manager role in relation to the physician role within the healthcare organisation. This interpretation is supported by other studies. A study of physicians becoming clinical managers [29] revealed how these managers regarded the manager position as a position for performing medical leadership rather than management. Actually, they regarded management as being *solely *an administrative task, that is, a part of the job that interfered with their medical leadership or their clinical practice. Another investigation [30] showed that, regardless of the area of their original profession, healthcare managers tended to regard their original profession as more important, and they based their decision making on that profession rather than on their more recently developed professional roles as managers [30]. Clarity about manager role authority and accountability has been proposed as a requirement for performance and well-being at work [31]. This also accounts for acknowledgement and management of necessary uncertainties and ambiguities surrounding one's own role in relation to the roles of others, both vertically and laterally [32].

According to Abbot [13], jurisdiction is negotiated not only in the workplace arena, as studied here, but also in the public and the legal arena. The latter two arenas can be regarded as contexts in which the manager role in healthcare organisations is performed. In the public arena in Sweden, as well as in many other countries, there has been an intense debate concerning the managerial threat to physician professionalism. In the legal arena, the laws enacted in Sweden in 1983 and 1997, can, in line with this reasoning, be interpreted as attempts to decrease physician autonomy by increasing managerial jurisdiction. However, our results support the findings of Salter [18] and indicate that these laws have failed in this attempt. Many of the CEOs described a relatively passive manager role, not as actors, but rather as "re-actors" in relation to physicians, in various ways trying to adjust their managerial strategies to physicians' behaviour. However, even though our results show that management of physicians was rather limited, the CEOs stated that physicians themselves felt that they were "over managed." This was also found in a study conducted in Spain, where it was observed that hospital physicians attributed the highest level of power to the managers, whereas the managers themselves felt that the greatest amount of power was held by physicians [33]. This apparent contradiction needs to be further investigated.

### Methodological considerations

A strength of this study is that all the CEOs of the 20 county councils in Sweden were included, and the response rate was high (90%). This means that the results probably reflect a representative variety of aspects of the research issue [22] and can be considered to have high credibility. Generalisability can be claimed only regarding Sweden, although the findings are no doubt also relevant for the situation in many other Western countries where the manager role is formally separated from the medical profession. The data were collected within the realm of a comprehensive project focused mainly on other aspects, which means that there was less chance of socially desirable answers, and the fact that this additional topic yielded such strong responses also supports the validity of the data. Further strengths include the following: the richness of the material, the openness of the interview participants, the involvement of two interviewers with experience of working with management issues, meetings held between the project group and the interviewers on several occasions during the data collection period, and three researchers with different scientific and professional backgrounds participating in the data analyses. In the analysis process, we chose to relate empirical data to prior and emerging theory, which enabled us to interpret data more systematically, thereby increasing the validity and degree of explanation of our results. Using only descriptive methods in data analyses tends to undervalue the importance of setting (organisation) and power (context) [27,28].

A possible limitation of our investigation is that we had no information about what type of professional training the CEOs who were not physicians had undergone. However, the focus of our study was the manger role, regardless of the basic profession of the participating CEOs, and thus potential differences between these managers in relation to their original profession was not within the scope of our analysis.

Our study was part of a larger Swedish study concerning management of sickness certification issues. Many physicians regard sickness certification problematic [24,34] and report that they lack management concerning this task [35]. The current results suggest a more complex picture, as managers seem to struggle to take their manager role in relation to the medical profession. More research on this topic is, therefore, warranted.

## Conclusions

This study shows that many top managers in Swedish healthcare seemed to regard the manager role in their organisations as weak in relation to the medical profession. This weakness is in sharp contrast to the increase in formal power given to healthcare managers in Sweden during the last decades. In this respect, an informal social structure exists in parallel to the formal organisational structure of the healthcare organisation. Many managers seem to struggle with this discrepancy between formal and informal social structures, and are unclear on how to take their managerial role in relation to the medical profession, irrespective of whether they themselves are or are not physicians. This implies that problems in the manager-physician relationship, which have been described in several studies, need to be discussed and investigated not only as an issue of relationships between managers and practicing physicians, but also in terms of managers' own role taking.

Our results also show that managers use strategies to manage physicians that seem to weaken the manager role in the organisation. This seems to reflect pragmatic behaviour on behalf of the managers. To maintain good relations and avoid conflicts is, of course, of great importance for managers at all levels in everyday practice. Physicians constitute a core group in healthcare, and being pragmatic is a way to maintain a certain degree of control in relation to a strong and important profession. However, studies indicate that lack of clarity concerning manager role authority and responsibility may have negative consequences—not only for the working conditions of managers, physicians, and other healthcare professionals, but also for the quality of care. It is therefore important that managers at all levels in the healthcare organisation discuss how the manager role is to be taken in relation to the medical profession.

## Competing interests

The authors declare that they have no competing interests.

## Authors' contributions

All three authors were involved in the design of the study, analysis and interpretation of the data, as well as drafting and reviewing of the manuscript. All authors read and approved the final manuscript.

## Pre-publication history

The pre-publication history for this paper can be accessed here:

http://www.biomedcentral.com/1472-6963/10/271/prepub

## References

[B1] Firth-Cozens J Mowbray D Leadership and the quality of care Quality in Health Care 2001 10 Suppl II ii3 ii7 11700372 10.1136/qhc.0100003.. PMC1765760

[B2] Smith R Why are doctors so unhappy? BMJ 2001 322 1073 1074 10.1136/bmj.322.7294.1073 11337419 PMC1120219

[B3] Nash DB Doctors and managers: mind the gap British Medical Journal 2003 326 652 653 10.1136/bmj.326.7390.652/a 12649246 PMC1125546

[B4] Brommels M Sjörs G, Lindqvist L, Varlsson I-M Solister i kör "Så är det bara ..." Om läkares ledarskap, yrkesroller och arbetsvillkor vid Akademiska sjukhuset 1998 Uppsala: Akademiska sjukhuset

[B5] Treasure T Redefining leadership in health care BMJ 2001 323 1263 1264 10.1136/bmj.323.7324.1263 11731375 PMC1121737

[B6] Kaissi A Manager-physician relationships: an organizational theory perspective Health Care Managment 2005 24 2 165 176 10.1097/00126450-200504000-00010 15923929

[B7] Shortell SM Waters T Clark KW Budetti PP Physicians as double agents: Maintaining trust in an area of multiple accountabilities Jounal of the American Medical Association 1998 280 12 1102 1108 10.1001/jama.280.12.1102 9757863

[B8] Edwards N Doctors and managers: poor relationships may be damaging patients - what can be done? Quality and Safety in Health Care 2003 12 21 14645744 10.1136/qhc.12.suppl_1.i21 PMC1765767

[B9] Davies H Hodges C Rundall T Views of doctors and managers on the doctor-manager relationship in the NHS BMJ 2003 326 7390 626 628 10.1136/bmj.326.7390.626 12649235 PMC151973

[B10] Rivers PA Woodard B Munchus G Organizational power and conflict regarding the hospital-physician relationship: symbolic or substantive? Health Services Management Research 1997 10 91 106 10168964 10.1177/095148489701000110

[B11] Rundall T Davies H Hodges C Doctor-manager relationships in the United States and the United Kingdom J Healthc Manag 2004 49 4 251 268 discussion 268-270 15328659

[B12] Danermark B Explaining Society. An Introduction to Critical Realism in the Social Sciences 2001 London: Taylor & Francis

[B13] Abbott A The system of professions 1988 London: The university of Chicago Press

[B14] Freidson E Professionalism. The Third Logic 2001 Chicago: University of Chicago Press

[B15] SOSFS Socialstyrelsens allmänna råd. Verksamhetschef inom hälso- och sjukvården (In Swedish) 1997 Socialstyrelsen 8

[B16] Whitley R Academic Knowledge and Work Jurisdiction in Management Organization Studies 1995 16 81 105 10.1177/017084069501600105

[B17] Grey C Management as a Technical Practice: Professonalization or Responsibilization? Systems Practice 1997 10 6 703 725 10.1007/BF02557921

[B18] Salter B Governing UK medical performance: A struggle for policy dominance Health Policy 2007 82 263 275 10.1016/j.healthpol.2006.10.004 17109988

[B19] Bannink D Kuipers S Lantink T (Eds.) Reform in Europe: braking the barriers in government 2006

[B20] Mintzberg H Toward healthier hospitals Health Care Manage Rev 1997 22 4 9 18 9358257 10.1097/00004010-199710000-00005

[B21] Yukl G Leadership in organizations 2002 5 New Jersey: Prentice Hall

[B22] Patton M Qualitative Research and Evaluation Methods 2002 3 London: Sage

[B23] Landstingsanställd personal 2006 http://www.skl.se/web/Landstingsanstalld_personal_2006_1_1.aspx

[B24] Alexanderson K Norlund A Sickness absence - causes, consequences, and physicians' sickness certification practice. A systematic literature review by the Swedish Council on Technology Assessment in Health Care Scandinavian Journal of Public Health 2004 32 Supplement 63 1 263 15513647 10.1080/14034950410003826

[B25] Corbin J Strauss A Basics of Qualitative Research. Tecniques and Procedures for Developing Grounded Theory 2008 3 Tousand Oaks: Sage

[B26] Graneheim UH Lundman B Qualitative content analysis in nursing research: concepts, procedures and measures to achieve trustworthiness Nurse Educ Today 2004 24 2 105 112 10.1016/j.nedt.2003.10.001 14769454

[B27] Layder D New strategies in social research 1993 Cambridge: Blackwell

[B28] Layder D Sociological practice. Linking theory and social research 1998 London: Sage

[B29] Öfverström H The step to management. Doctors as clinical managers. (In Swedish) 2008 Gothenburg University, School of economics

[B30] Lindholm M Udén G Råstam L Management from four different perspectives Journal of Nursing Management 1998 7 101 111 10.1046/j.1365-2834.1999.00035.x 10373849

[B31] Jaques E Requisite organisation. A total system for effective managerial organisation and managerial leadership for the 21st century 1996 Arlington VA: Cason Hall

[B32] Huffington C James K Armstrong D What is the emotional cost of distributed leadership? Conference paper, unpublished 2003

[B33] Salvadores P Schneider J Zubero I Theoretical and perceived balance of power inside Spanish public hospitals BMC Health Service Research 2001 1 1 9 10.1186/1472-6963-1-9 PMC56903 11574049

[B34] Hussey S Hoddinott P Wilson P Dowell J Barbour R Sickness certification system in the United Kingdom: qualitative study of views of general practitioners in Scotland BMJ 2004 328 7431 88 10.1136/bmj.37949.656389.EE 14691065 PMC314050

[B35] von Knorring M Sundberg L Löfgren A Alexanderson K Problems in sickness certification of patients: A qualitative study on views of 26 physicians in Sweden Scandinavian Journal of Primary Health Care 2008 26 1 22 28 10.1080/02813430701747695 18297559 PMC3406623

